# Implementing Direct Access to Low-Dose Computed Tomography in General Practice—Method, Adaption and Outcome

**DOI:** 10.1371/journal.pone.0112162

**Published:** 2014-11-10

**Authors:** Louise Mahncke Guldbrandt, Torben Riis Rasmussen, Finn Rasmussen, Peter Vedsted

**Affiliations:** 1 Research Centre for Cancer Diagnosis in Primary Care, Research Unit for General Practice, Aarhus University, Aarhus, Denmark; 2 Section for General Medical Practice, Department of Public Health, Aarhus University, Aarhus, Denmark; 3 Department of Respiratory Diseases and Allergy, Aarhus University Hospital, Aarhus, Denmark; 4 Department of Radiology, Aarhus University Hospital, Aarhus, Denmark; University of Oxford, United Kingdom

## Abstract

**Background:**

Early detection of lung cancer is crucial as the prognosis depends on the disease stage. Chest radiographs has been the principal diagnostic tool for general practitioners (GPs), but implies a potential risk of false negative results, while computed tomography (CT) has a higher sensitivity. The aim of this study was to describe the implementation of direct access to low-dose CT (LDCT) from general practice.

**Methods:**

We conducted a cohort study nested in a randomised study. A total of 119 general practices with 266 GPs were randomised into two groups. Intervention GPs were offered direct access to chest LDCT combined with a Continuing Medical Education (CME) meeting on lung cancer diagnosis.

**Results:**

During a 19-month period, 648 patients were referred to LDCT (0.18/1000 adults on GP list/month). Half of the patients needed further diagnostic work-up, and 15 (2.3%, 95% CI: 1.3–3.8%) of the patients had lung cancer; 60% (95% CI: 32.3–83.7%) in a localised stage. The GP referral rate was 61% higher for CME participants compared to non-participants.

**Conclusion:**

Of all patients referred to LDCT, 2.3% were diagnosed with lung cancer with a favourable stage distribution. Half of the referred patients needed additional diagnostic work-up. There was an association between participation in CME and use of CT scan. The proportion of cancers diagnosed through the usual fast-track evaluation was 2.2 times higher in the group of CME-participating GPs. The question remains if primary care case-finding with LDCT is a better option for patients having signs and symptoms indicating lung cancer than a screening program. Whether open access to LDCT may provide earlier diagnosis of lung cancer is yet unknown and a randomised trial is required to assess any effect on outcome.

**Trial Registration:**

Clinicaltrials.gov NCT01527214

## Background

Lung cancer is the leading cause of cancer death among men on a global basis. For women, it is the second leading cause of cancer death [1]. Annually, 4400 patients with lung cancer are diagnosed in Denmark [2]. Disease stage at diagnosis is an important prognostic factor as an advanced stage reduces the opportunity for curative treatment. Therefore, it is crucial to reduce the proportion of lung cancers diagnosed at an advanced stage; in Denmark, advanced-stage cancers account for 70% of all new lung cancers.

In order to reduce the time interval from the first presentation to the healthcare system until treatment, Denmark introduced a fast-track referral program for cancer in 2008 [3], [4]. In this program, Danish general practitioners (GPs) can refer patients with “reasonable suspicion” of lung cancer to a fast-track evaluation, a maximum of 72 hours waiting time. Unfortunately, only 25% of Danish lung cancer patients are referred and diagnosed through this fast-track pathway, which is similar to the level of the UK [5]–[7]. Studies indicate that lung cancer patients have several pre-referral consultations in primary care [8], [9]. This could be based on the fact that many lung cancer patients seem to present with unspecific, vague or low-risk-but-not-no-risk symptoms [10]. This implies that GPs needs additional tools than the fast-track in order to ensure early diagnosis of lung cancer. The answer could be direct access to a sensitive diagnostic investigation.

The principal diagnostic tool available for the GPs has for many years been a chest radiograph. However, since about 20% of all lung cancer patients have normal radiographs before diagnosis [11]–[13], a false negative radiograph may postpone the diagnosis [11]. Thus, an open direct access should perhaps be combined with a technological update in use of Computed Tomography (CT) technology.

In screening trials, low-dose CT is used under the presumptions that 1) lung cancer presents as non-calcified nodules, 2) low-dose CT accurately detects these nodules, and 3) detection of early-stage disease improves prognosis. From screening studies in high risk patients, we already know that approx. 27% of the first-round screened patients needed follow-up scans [14], [15]. On the other hand, we do not know the same figures for symptomatic patients visiting their GP. Likewise, we do not know whether the GPs will use direct access to CT when offered the opportunity or (if positive) which patients they will refer. Such data should be available before chest LDCTs are introduced as a routine test for patients with respiratory symptoms.

The aim of this study was to describe the usage and outcome of a technological and organisational upgrade in the form of a brief GP update and implementing direct access to chest CT from general practice for patients with respiratory symptoms. Furthermore, to analyse the association between participating in the update, use of CT scans and referrals for lung cancer suspicion.

## Methods

### Design

We conducted a cohort study nested in a randomised controlled trial. A random group of GPs were offered a technological upgrade consisting of direct access to chest LDCT combined with a simple Continuing Medical Education (CME) meeting on lung cancer diagnosis.

### Setting

The study took place in a large catchment area around Aarhus University Hospital in the Central Denmark Region during 19 months (November 2011 to June 2013).

Denmark has a tax-financed healthcare system with free access to medical advice and treatment in general practices and hospitals. GPs act as gatekeepers to specialized investigations and hospitals referrals.

Before November in 2011, the GPs in the area had three diagnostic work-up possibilities for patients with respiratory symptoms. They could either refer to 1) a chest radiograph 2) the Department of Pulmonary Medicine within normal waiting list or 3) a fast-track pathway. A valid indication for fast track was either an abnormal chest radiograph or certain symptoms (e.g. haemoptysis of >one week’s duration or persistent coughing >four weeks). The GPs were not allowed to refer directly to a CT.

### Participants

All GPs referring to the Department of Pulmonary Medicine. A total of 266 GPs organised into 119 general practices, were randomised into two groups. The unit of randomisation was the practice address. The randomisation was performed by a Data Manager using Stata 12.0. The 119 practices were allocated a random number between zero and one and then listed from lowest to highest value. The top 60 practice addresses formed the intervention group. In this paper, we include only the intervention group.

### Intervention

Six times within an 3-month period, the intervention GPs were informed about the opportunity to refer their patients to a direct chest CT. The letters included information concerning the referral procedures and specific indications for CT requests. These indications embraced a wide range of concerns; the only exception was patients who met the indication for a fast-track referral. The idea was to let the GPs substitute the radiograph with a chest LDCT when wanting to rule out lung cancer.

The GPs were also invited to participate in one of eight offered 1-hour small-group-based CME meetings. The meetings were held during the initial two months. The content of the meeting focused on state-of-the-art knowledge on earlier detection of lung cancer. Algorithms for positive predictive values in primary care were used [10], [16]. In addition, participants received information about the CTs, how to use them and how to interpret the reports.

### Chest CT, review and lung cancer diagnosis

The Department of Radiology, Aarhus University Hospital, carried out the CTs. Scans were performed on a Brilliance 64 CT Scanner by Philips with a beam collimation of 64×0.625, 2 mm slice thickness, 1 mm increment, 1 pitch and a rotation time of 0.75 s. The effective radiation dose (Monte Carlo simulation program CT-Expo v. 2.1) was 2–3 mSv. Intravenous contrast medium was not administered. The time limit from referral to performed CT was a maximum of two working days.

The CT report was made by three sub-specialised radiologists. The day after the scan, the report combined with the patient’s medical history resulted in a recommendation drawn up at a conference between radiologists and chest physicians. This recommendation was forwarded electronically to the GP, who was responsible for informing the patient of the results and referring the patient to further diagnostic work-up if necessary.

If lung nodules (4–10 mm), which could not be categorised as benign, were detected, the GP was responsible for referring the patient to a follow-up program (3, 6 or 12 months after the first scan) based on characteristics of the identified nodules [17]. The follow-up program was decided by the chest physicians.

If the CT scan revealed any suspicion of lung cancer, the patients were referred through the fast track to standard diagnostic work-up at the Department of Pulmonary Medicine by the GP. This included contrast enhanced multi detection CT (including PET/CT if surgery was an option). Furthermore, histologic/cytologic diagnosis was obtained by the least invasive method, which was usually either bronchoscopy with biopsies, fine needle aspiration (FNA) in association with endoscopic ultrasound or endobronchial ultrasound, or transthoracic FNA. The final staging was decided by a multi-disciplinary team based on clinical (cTNM) information. The lung cancers were staged according to the 7th TNM Classification of Malignant Tumors [18]. Early stage cancers were defined as stage I–IIB. Early stage patients were offered surgical resection according to Danish guidelines.

### Sample size

The sample size was calculated for the randomised trial and the numbers of GPs needed in the intervention arm was guided by the calculation. In 2008, half of the Danish lung patients waited 34 days or more (the median) from first presentation to primary care until diagnosis of lung cancer [19]. We hoped to be able to show a decrease in the diagnostic interval to a level where only 25% of the patients had to wait for 34 days or more. Thus, the proportion waiting 34 days or more should be halved. With a one-sided alpha of 5% and a power of 80%, we had to include 54 lung cancer patients in each arm with a 1∶1 randomisation. It can be assumed that lung cancer patients are randomly distributed among GPs. There could, however, be a higher incidence of cancer in some areas with many smokers and in practices with many elderly patients. To account for an unknown intra-cluster correlation coefficient (ICC), we counted on a design effect of 1.25 [20]. Given the design effect, we had to include a total of 54*2*1.25 = 135 lung cancer patients with questionnaire data and GP involvement in the diagnosis.

### Outcome variables

Primary outcomes were characteristics of patients referred and GP variation in use, while secondary outcomes were amount of diagnostic work-up needed and cancer incidence. Finally, we examined the use of the fast-track referral option for suspected lung cancer and the proportion of lung cancer (the positive predictive value (PPV)) in order to evaluate the possible effect of the CME on this aspect.

### Data

Based on the GPs’ referral notes, we obtained data on the patients symptoms, known diseases and smoking history. We obtained the medical records resulting from completed CT scans, including the consensus evaluation between radiologist and pulmonary physician.

The Danish Lung Cancer Register (DLCR) was used for information on subsequent diagnosis of lung cancer (International Classification of Diseases 10: C34.0-9). The DLCR was established in 2001 as a national data­base. Since 2003, the registered data have covered more than 90% of all lung cancer cases in Denmark [21].

Patients referred to fast-track evaluation for lung cancer are coded DZ 03.1B (lung cancer observation). This code, combined with a unique GP number, gave information about referral to the fast-track pathway.

The Danish Cancer Registry (DCR) was used to obtain information about previous cancer (except non-melanoma skin cancer (C44)). The registry contains information about Danish cancer patients, their date of diagnosis and tumour characteristics. Since 1987, reporting to the DCR has been mandatory [22].

We used the Danish Deprivation Index (DADI) to gather information about deprivation rates in the different GP clinics. The index consists of eight variables resulting in a value number between 10 and 100; the higher the number, the greater the extent of deprivation in the practice population. The variables used are: (i) Proportion of adults aged 20–59 with no employment, (ii) proportion of adults aged 25–59 with no professional education, (iii) proportion of adults aged 25–59 with low income, (iv) proportion of adults aged 18–59 receiving public welfare payments (transfer income or social benefits), (v) proportion of children from parents with no education and no professional skills, (vi) proportion of immigrants, (vii) proportion of adults aged 30+ living alone and (viii) proportion of adults aged 70+ with low income ( =  the lowest national quartile).

The Health Service Registry was used to gather information about GP list size and age/gender distribution of the patients listed with the GP [23].

The Danish civil registration number, a unique personal identification number, was used to link registers [24].

### Statistical analyses

Patient characteristics were described and duration of symptoms was calculated as medians with interquartile intervals (IQI). GP groups were compared using the Wilcoxon’s rank-sum test for ordinal or continuous data or Pearsons χ^2^ test for unordered or dichotomous categorical data.

Referral rates were calculated based on number of patients referred by the GP per project month per list size (patients aged 25 years and above). We used indirect sex-age standardisation to compare the referral rates between CME-attending GPs and non-attending GPs. We used the CME-attending GPs as the standard population and calculated the referral rates for the patients listed with the GPs in 10-years age groups (25–34, 35–44, etc.). These expected rates were then applied to the non-attending GP list. We calculated the standardized referral rate ratio as number of referrals divided by expected numbers if the age-sex specific rates were the same as those of the standard population. The age-sex referral rate was then obtained by multiplying the referral rate ratio by the crude referral rate of the standard population.

Data were analysed using the statistical software Stata 12.0 (StataCorp LP, TX, USA).

The protocol for the randomised trial and supporting TREND checklist for this study are available as supporting information; see Checklist S1 and Protocol S1.

### Ethics

The study was approved by the Danish Data Protection Agency (ref.no: 2011-41-6872) and the Danish Health and Medicines Authority (ref.no: 7-604-04-2/357/KWH). According to the Research Ethics Committee of the Central Denmark Region, the Danish Act on Research Ethics Review of Health Research Projects did not apply to this project (ref.no: 118/2011) as CT is already a widely used technology.

## Results

### Patients referred

During the study period of 19 months, 649 patients were referred from general practice to direct CT. One patient (0.15%) did not turn up to the scan, resulting in 648 performed CTs. The mean age of scanned patients was 62.1 years (Standard Deviation (SD): 12.3, range: 21–95 years) (Table 1). The mean number of pack years for all smokers (current and former) was 34.5 (SD: 1.4, range: 2–100), and 87 (13.4%, 95% CI: 10.9–16.3%) had never smoked. The most prominent symptom was coughing (78.2%, 95% CI: 74.9–81.4%). The duration of symptoms varied from a median of 1.5 weeks (haemoptysis) to a median of 8.0 weeks (coughing) (Table 2). For 124 (19.1%, 95% CI: 16.2–22.4%) patients, a known lung disease (mostly COPD) was stated in the referral letter (Table 1).

**Table 1:** Clinical characteristics of the 648 patients referred to direct CT scan from general practice.

	N	(%)^1^	Mean	(95% CI)
Gender:				
* Male*	314	(48.5)		
* Female*	334	(51.5)		
Age all	648		62.1	(61.2–63.1)
Age groups:				
* 20–45 yr*	62	(9.6)		
* 46–65 yr*	320	(49.3)		
* 66–95 yr*	266	(41.1)		
Smoking status:				
* Never*	87	(13.8)		
* Current*	257	(40.7)		
* Former*	131	(20.7)		
* Missing*	157	(24.8)		
Pack years:				
* All smokers*	133		34.5	(31.6–37.3)
* Current*	89		38.1	(34.6–41.7)
* Former*	44		27.1	(23.0–31.2)
Known lung disease:				
* All*	124	(19.1)		
*Previous cancer^2^:*				
* ≥10 years*	24	(3.7)		
* <10 years*	34	(5.2)		

^1^Of all patients.

^2^Listed in DCR before study start (either ≥10 years before study or within 10 years).

**Table 2:** Symptoms written on referral letters of the 648 patients referred to direct CT scan from general practice.

	N	(%)^1^	Median	(IQI^2^, min-max)
Focal symptoms:				
* Cough*	507	(78.2)		
* Duration^3^*	309		8	(6–12, 1–104)
* Dyspnoea*	170	(26, 2)		
* Duration^3^*	76		8	(5.5–12, 1–103)
* Expectoration*	165	(25.5)		
* Duration^3^*	69		8	(4–12, 1–104)
* Thorax pain*	90	(13.9)		
* Duration^3^*	46		4.5	(4–12, 1–52)
* Haemoptysis*	51	(7.9)		
* Duration^3^*	18		1.5	(1–3, 0–12)
* Hoarseness*	25	(3.9)		
* Duration^3^*	10		8	(4–6, 2–40)
General symptoms:				
* Fatigue*	85	(13.1)		
* Duration^3^*	42		6	(4–12, −26)
* Weight loss*	79	(12.2)		
* Duration^3^*	45		8	(4–12, 1–52)
* Impaired general condition*	48	(7.4)		
* Duration^3^*	18		4	(4–6, 2–40)

^1^Of all patients.

^2^Inter quartile interval.

^3^Duration in weeks. Some missing data.

### GP participants

**Figure 1:**
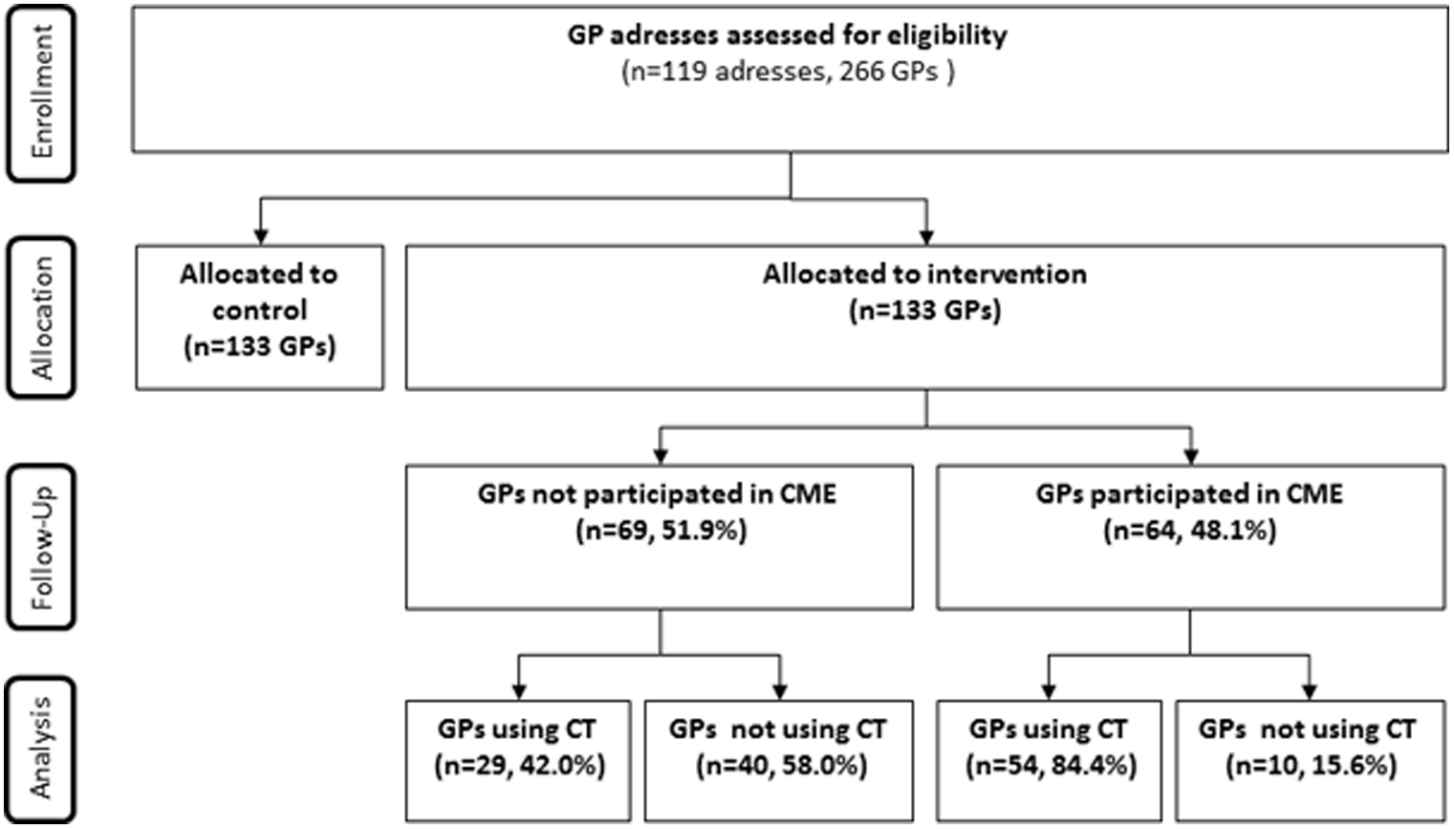
Participants (GPs) flow.

A total of 133 GPs had access to direct CT (Figure 1). The possibility was used by 91 (68.4%, 95% CI: 59.8–76.2%) of the GPs (Table 3). The highest absolute number of CT requests from a single GP was 40 (2 per project month), whereas most GPs referred two patients during the study period (median: 2.0, IQI: 0–5) (Table 3).

**Table 3:** The characteristics of the GPs in the intervention group, their use of CT and participation in CME.

	All		CT not used		CT used		p-value	CME participant		Not CME participant		p-value
All GPs, No (%)	133		42	(31.6)	91	(68.4)		64	(48.1)	69	(51.9)	
Gender:												
Female, No (%)	65	(48.9)	18	(27.7)	47	(72.3)		31	(47.7)	34	(52.3)	
Male, No (%)	68	(51.1)	24	(35.3)	44	(63.7)	0.358	33	(48.5)	35	(51.5)	1.000
Age, mean (range)	53.6	(35–68)	54.2	(38–66)	53.4	(35–68)	0.613	54.2	(39–68)	53.1	(35–66)	0.456
Practice type:												
One GP, No (%)	21	(35.0)	11	(52.4)	10	(47.6)		8	(38.0)	13	(62.0)	
Two or more GPs, No (%)	39	(65.0)	11	(28.2)	28	(71.8)	0.090	24	(61.5)	15	(38.5)	0.107
Practice list size/GP Median (range)	1008	(585–2780)	997	(585–1503)	1012	(585–2780)	0.794	1056	(639–2780)	963	(585–1507)	0.080
Number of patients scanned:												
Per GP, Median (IQI)	2	(0–5)	0	(0–0)	3	(2–8)	**<0.001**	3	(1–9)	1	(0–3)	**<0.001**
Per practice, Median (IQI)^1^	6	(1–22)	0	(0–3)	17	(4–24)	**<0.001**	17	(4–23)	3	(0–4)	**<0.001**
Referral rate^2^, Median (IQI)	0.10	(0–0.30)	0	(0–0)	0.18	(0.09–0.45)	**<0.001**	0.15	(0.05–0.59)	0.05	(0–0.18)	**<0.001**
DADI^3^, Median (IQI)	25.4	(20.5–31.6)	26.5	(19.5–30.8)	25	(20.8–32.0)	0.947	24.1	(19.6–32.8)	26.0	(20.5–30.9)	0.920
CME:												
Participants	64	(48.1)	11	(17.2)	53	(82.8)	**<0.001**					
Age-sex adjusted referral rate^4^:									1	0.39		

^1^If one GP in a clinic has participated in CME, all GPs in that clinic will count as CME participants.

^2^Referral rate: patient referred/1000 patients in GP list (patients ≥25 years)/project months.

^3^Danish Deprivation Index (min: 10- max: 100).

4 Referral rate adjusted for age and gender distribution in GP list (patients ≥25 years).

When we excluded the GPs who did not use the possibility of direct CT, the unadjusted GP referral rate was 0.18 per 1000 patients (≥25 years of age) per month.

There was no difference in GP age, gender, type of clinic (solo or more GPs together), list size or levels of deprivation in relation to the use of CT scans.

In total, 64 (48.1%, 95% CI: 39.4–56.9%) of the GPs participated in the CME meeting. The referral rate to direct CT was statistically significantly higher among GPs working in a clinic with one or more CME-participating GPs. When adjusting for age, gender and list size, the referral rate was 61% higher (95% CI: 54–66%) for GPs working in a clinic with one or more CME-participating GPs than the referral rate for non-participating GPs.

The study GPs referred 335 patients to the lung cancer fast-track during the study period, and this resulted in 33 lung cancer diagnoses (PPV 10.2%, 95% CI: 7.2–13.9%). The stage distribution was as follows 8 (23.5%, 95% CI: 10.7–41.2%) were in early stage and 26 (76.5%, 95% CI: 58.8–89.3%) with advanced disease. The unadjusted referral rate to fast-track was 0.13 per 1000 adults listed with the GP per month (95% CI: 0.09–0.20). The referral rate was 0.13 (95% CI: 0.09–0.19) for CME-participating GPs compared with 0.14 (95% CI: 0.09–0.20) for non-participating GPs (p-value: 0.503). The PPV for lung cancer diagnosis as a result of referral to a fast-track lung cancer pathway was 13.3% (95% CI: 8.7–19.1%) for CME-participating GPs and 6.1% (95% CI: 3.0–11.0%) for non-participating GPs (p-value: 0.027), which is equivalent to a 2.2 higher PPV.

### Evaluation and conclusions

Of the 648 patients who underwent CT, 234 (36.1%, 95% CI: 32.0–40.0%) patients had a normal scan (Table 4), while lung nodules were found in 147 patients (22.7%, 95% CI: 19.5–26.1%). Cancer suspicion was raised in 84 (13.0%, 95% CI: 10.5–15.8%) of the scans, and suspicion of other lung diseases was raised in 200 (30.9%, 95% CI: 27.3–34.6%). For 301 (46.5%, 95% CI: 42.6–50.4%) patients, no further diagnostic work-up was needed.

**Table 4:** The evaluation of the 648 CT scans performed during the study period.

	Number	(%) of allscans
All scans:	648	(100.0)
**Evaluation:**		
Abnormal scan:	414	(63.9)
* Nodules*	147	(22.7)
Cancer suspicion:		
* All*	84	(13.0)
* Lung*	71	(11.0)
* Breast*	6	(0.9)
* Liver*	1	(0.2)
* Mesothelioma*	3	(0.4)
* Renal*	2	(0.3)
Occult	1	(0.2)
Lung disease suspicion:		
* All*	200	(30.9)
* Pneumonia*	81	(12.5)
* Pulmonary fibrosis*	69	(10.6)
* Emphysema*	44	(6.8)
* Bronchiectasis*	19	(2.9)
* Tuberculosis*	6	(0.9)
Suspicion of other diseases:		
* All*	119	(18.4)
* Enlarged lymph nodes*	52	(8.1)
* Liver^1^*	32	(4.9)
* Bone^2^*	21	(3.2)
* Biliary^3^*	9	(1.4)
* Pancreas* 4	5	(0.8)

^1^Lever disease: all focal changes; cysts/metastases observation.

^2^Bone: 13 fracture obs., 1 Mb Bechterew obs., 3 metasteses obs.

^3^Billiary: All cholecystelithiasis obs.

4 Pancreas: 3 chronic pancreatitis.

A total of 177 (27.3%, 95% CI: 23.9–30.9%) patients received a referral to the Department of Pulmonary Medicine for further diagnostic work-up. Suspicion of disease outside the lungs was raised in 38 (5.9%, 95% CI: 4.2–8.0%) patients (Table 5).

**Table 5:** The conclusion and diagnosis of the 648 CT scans performed during the study period.

	Number	(%) of all scans
All scans:	648	(100.0)
**Conclusions:**		
* No further*	301	(46.5)
* Pulmonary medicine*	177	(27.3)
* CT scan (3 month after)*	84	(13.0)
* CT scan (6 month after)*	23	(3.5)
* CT scan (12 month after)*	51	(7.9)
* Other department*	38	(5.9)
* Treatment by GP*	15	(2.3)
**Diseases lung:**	**Number all/new** **diagnoses^1^**	
* All*	93	(14.4)
* Tuberculosis*	5/5	(0.8/0.8)
* Sarcoidosis*	8/7	(1.2/1.1)
* Interstitiel*	17/17	(2.6/2.6)
* Emphysema*	44/29	(6.8/4.5)
* Bronchiectasis*	19/19	(2.9/2.9)
**Lung cancer:**		
* All*	15	(2.3)
* NSCLC*	15	(100.0)^2^
* Local*	9	(60.0)^2^
* Metastatic*	6	(40.0)^2^
***Other cancer:***		
* All*	8	(1.2)

^1^All lung disease diagnoses were new, except for 15 patients with emphysema and one patient with sarcoidosis (they had the diagnosis before the CT).

^2^Of all lung cancers diagnosed in the study.

### Definitive diagnoses made from baseline scans

Thirty (4.6%, 95% CI: 3.1–6.5%) patients were diagnosed with a severe lung disease (tuberculosis, sarcoidosis or interstitial lung disease). Fifteen (2.3%, 95% CI: 1.3–3.8%) had a non-small cell lung cancer (NSCLC) and none had a small cell lung cancer (SCLC). Stage distribution was as follows: nine (60%, 95% CI: 32.3–83.7%) in early stage and six (40%, 95% CI: 16.3–67.7%) with advanced disease. Six (40.0%, 95% CI: 16.3–67.7%) were stage I tumours. Eight (1.2%) other cancers were diagnosed (three breast cancers, two lymphomas, one rectal cancer, one hepatocellular carcinoma and one mesothelioma).

## Discussion

### Main results

During the study period, 648 patients were referred to a direct LDCT. The most prominent symptom was coughing with a median duration of two months. Half of the patients needed further diagnostic work-up and 2.3% had lung cancer; 60% in early stage.

Two thirds of the GPs used the direct access to LDCT. CME-participating GPs had a 61% higher CT referral rate than non-participating GPs. CME participation was not associated with increased use of lung cancer fast-track pathways, but was, however, associated with a more than doubled positive predictive value.

### Strength and limitations

A major strength of this study is the well-defined study population of a considerable size of patients. The data obtained from the referral letters and the CT records were complete as were data on GP participation in the CME on lung cancer.

However, a limitation is that we have no knowledge about the kind of diagnostic tool (e.g. plain chest film or fast-track) applied by the GP if (s)he had not had the opportunity of referral to direct CT scan.

The reported results are based on the baseline CT scan. A follow-up study is needed to gain information on lung cancers diagnosed from the repetitive CTs on nodule follow-up indications.

This study was not designed to answer whether a direct LDCT from general practice would reduce the mortality of lung cancer. A high proportion of the lung cancers diagnosed in this study were identified in an early stage, but this is not an advantage in itself. Early-state identification is beneficial only if the frequency of late-stage cancers is reduced, and this will be analysed in a randomised trial including all lung cancers in the study period.

The present study utilised low-dose CT as the diagnostic tool. For lung cancer, CT has a high sensitivity, but a lower specificity. This implies that the method involves risk of patient distress because of a relative high number of false positive scans. Furthermore, a widespread concern is the risk of cancer secondary to radiation from the low-dose CTs and subsequent imaging used to evaluate positive screens. A US study from 2013 addresses this problem in connection to low-dose CT screening studies [25]. Based on epidemiological data on radiation exposure they calculate that if assuming annual low-dose CT from age 55 to age 74 (20 scans), the lifetime attributable risk of lung cancer mortality is estimated to be 0.07% for males and 0.14 for females. One single low-dose CT utilizes not even half of the total annual radiation exposure from natural and human made sources. In addition, the group of patients referred to a low-dose CT may be among those with a higher risk of having lung cancer or other important diseases and the small radiation dose may contribute only very little to the other risks these patients face.

### Generalisability

This Danish single setting with complete inclusion of patients holds the opportunity to generalise the study results to other settings in Denmark, possibly even to other countries in which general practice serves as the first line of healthcare.

### Comparison with other studies

In this study, symptomatic patients consulted general practice and the GP referred them to a direct CT scan; 2.3% of the patients were consequently diagnosed with lung cancer. In a US screening study (NLST) (2002–2004) including participants aged 55–74 with at least 30 pack-years [15], 1.1% had lung cancer at baseline. The authors reported 55% stage I cancers compared to 40% in our study. In the screening study, 27.9% of the patients needed follow-up scans. This is comparable to our numbers. Similar results were seen in the Danish randomized lung cancer CT screening trial (DLCST) (2004–2006) [26], which included participants aged 50–70 with at least 20 pack-years; 0.83% of the participants were diagnosed with lung cancers (53% in stage I).

Compared with the screening trials, our study had a wide and GP-based inclusion for referral. By limiting GP access to the CTs with specific criteria (e.g. smokers or age above 50 years), the proportion of lung cancers diagnosed in our study would probably have been higher. However, the non-limited access shows the actual use and outcome when direct access is implemented. The fact that we found 40% stage I cancers in symptomatic patient could be due to an increased awareness of early signs of cancer among GPs in combination with easy access to a direct test.

The frequency of lung cancer was lower among patients referred directly to LDCT than for those referred to the lung cancer fast-track pathway. This indicates that the patients referred to a direct CT are a subgroup of patients with less pronounced symptoms and thus with a lower risk that the symptoms were due to cancer. Patients with “low, but not no risk” may be the ones who most GPs find difficult to handle in primary care. This is also supported by the higher PPV for cancer in the fast-track pathway for CME-participating GPs. We cannot make any causal inference of the associations found as these may be due to comparison of simply two different groups of GPs. However, our results may also indicate an effect of the CME and a changed pattern in use of direct access to CT, which can only be evaluated in an experimental design.

A Danish study found that a strategy with straight-to-test to CT for patients in the lung cancer fast-track was associated with high levels of staff acceptability and a reduction of chest physician time per patient without changing the numbers of performed CTs [27]. This implies that GPs are able to use CTs in a reasonably way. This is, in this present study, supported by the low overall referral rate.

In terms of variation we found no association between GP characteristics (age, gender, type of clinic, list size or levels of deprivation) and the use of CTs. A review from Scotland concluded that variation in GP referral rates in general is largely unexplained [28]. The study suggests that GPs with an interest or training in a particular field had a higher referral rate in that specialty. This may be the reason for the higher referral rate among GPs who participated in the CME. However, we can make no causal inference as these findings may be related to selection bias.

### Conclusion

In a cohort study on direct CT referral from general practice, we found an overall referral rate of 0.10/1000 adults/month. Two-thirds of the GPs used the open access CT option. An association was found between participation in a lung cancer CME and direct referral to CT. An association was also found between GP participation in a CME on lung cancer diagnosis and a higher PPV of lung cancer when referring to the fast-track pathway compared to non-participating GPs.

Among patients referred to a CT, the proportion of lung cancers was 2.3%, 1.2% had other cancers and 14.4% had a non-malignant serious lung disease. The CTs resulted in 53.5% in need of additional diagnostic work-up or follow-up scans. Whether the open access to chest CT will result in earlier diagnosis and better prognosis of lung cancer is yet unknown, and a randomised trial is required to assess any effect on outcome. The results from the randomised trial are under preparation for publication and the authors have planned a two year follow-up on the 648 patients scanned in this study in regard to additional diagnoses as well as further diagnostic procedures. The question remains whether case-finding with LDCT in primary care is a better option for patients having signs and symptoms indicating lung cancer than a screening program. Furthermore, if low-dose CT screening is recommended, a consideration is whether a direct LDCT option from primary care should be implemented as well for patients who are not screened.

## Supporting Information

Checklist S1 **TREND checklist.**
(DOCX)

Protocol S1 **Trial protocol.**
(DOCX)
