# Supplementary material for: Implementing Direct Access to Low-Dose Computed Tomography in General Practice—Method, Adaption and Outcome
Source: PLoS One. 2014 Nov 10;9(11):e112162. doi: 10.1371/journal.pone.0112162 (PMC4226510; doi:10.1371/journal.pone.0112162)
Supplement: Protocol S1 — Trial protocol. (DOCX) [file pone.0112162.s002.docx]

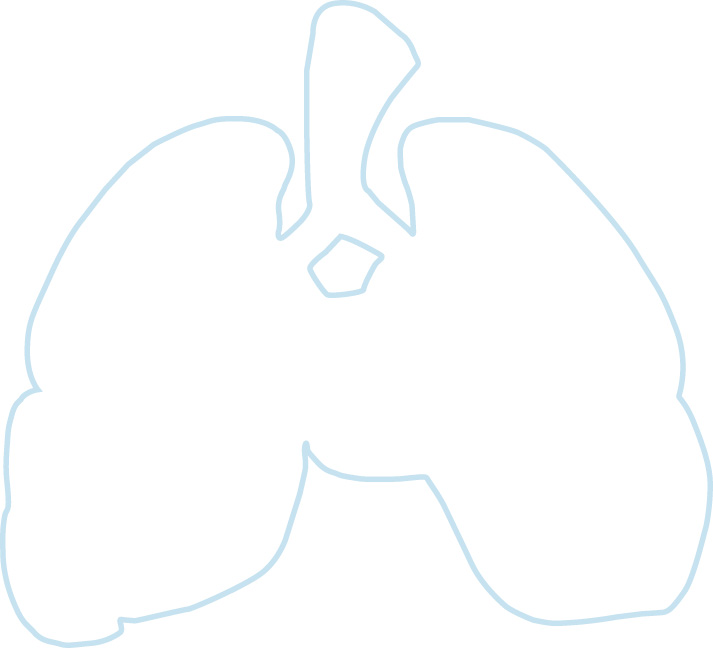


**Project description, Louise Mahncke, MD**

The effect of direct referral for fast CT scan in early lung cancer detection in general practice. Clinical, randomised trial.

| Institution: | The Research Unit for General Practice. The Danish Cancer Society and the Novo Nordisk Foundation Research Centre for Cancer Diagnosis in Primary Care – CaP |
| --- | --- |

| Research group: | Peter Vedsted, Professor, MD, PhD, CaP, The Research Unit for General Practice. |
| --- | --- |
|  | Peter Meldgaard, MD, PhD,  Department of Oncology, Aarhus University Hospital. |
|  | Torben Riis Rasmussen, MD, PhD,  Department of Pulmonary Medicine, Aarhus University Hospital. |
|  | Finn Rasmussen, DrMedSc,  Department of Radiology, Aarhus University Hospital. |

**1. Background**

Annually, 4,200 new cases of lung cancer are diagnosed in Denmark (1). The stage of the disease is an important prognostic factor as an advanced stage reduces the opportunity for surgical intervention and other curative treatment. If the diagnosis of primary lung cancer is established early (equalling low stage), there are increased opportunities for curative action and consequently improved prognosis. Lung cancer patients are among the cancer patients with the highest mortality (2). Thus, 5-year survival for stage IA is 60%, for stage IIIB 5% and for metastasis (stage IV) as low as 2 % (1). In Denmark, almost 70% of the patients have metastatic disease at the date of diagnosis which is more than in other Nordic countries (3). An early diagnosis is not only important for survival and treatment options, but also for palliative care (4).

For approx. 85% of the cases the primary investigation of lung cancer is conducted in general practice (5). Unfortunately, it tends to take relatively long time from the first visit to the general practitioner (GP) until the patients are diagnosed (6). We have previously shown that lung cancer patients have the longest delay in general practice. This is often because GPs have to wait for diagnostic examinations such as chest x-rays for this group of patients (7). To reduce this delay, a fast track referral for lung cancer was introduced in April 2008 (8).

More than 98% of the Danish citizens are registered with a GP. The average GP list size is approx. 1,600 patients. The GP functions as a gatekeeper to the rest of the health-care system, carrying out initial diagnostic investigations. When the GPs refer patients through the fast track, the majority of patients make their first visit to the Department of Pulmonary Medicine. After this visit, further investigation is initiated, which is often a CT scan of the chest and the upper abdomen. However, there is variation in this referral practice and in some areas the Department of Radiology is the primary access point. Whether differences in the organisation of the examination of lung cancer lead to significant differences in the proportion of positive findings and delay is unknown.

According to the guidelines, a GP has to consider lung cancer in people over 40 years with new respiratory or general symptoms lasting more than four weeks, or exacerbation of chronic respiratory symptoms. Relevant symptoms are symptoms such as unexplained cough, haemoptysis and constitutional symptoms such as weight loss, fatigue and loss of appetite. If the patient presents with such a clinical picture and if the GP has a “reasonable suspicion”, the patient should be referred to a chest x-ray or directly through the fast track. However, we lack knowledge about whether this is the most effective clinical strategy.

The frequency of alarm symptoms in the population is high. Nearly 15% of the adult population experienced warning symptoms during the last 12 months, and 6.5% coughed for more than six weeks (9). Many patients (e.g. chronically ill patients) will present with symptoms similar to symptoms of lung cancer, but most often due to benign causes. On average, a Danish GP only diagnoses one patient with lung cancer each year, and the GP will therefore get the clinical experience that when he refers a patient with alarm symptoms, the test often comes out negative, and this may make him fail to refer.

This also means that alarm symptoms have low positive predictive values (PPV). A case-control study found that all symptoms shown to be associated with lung cancer had PPVs of less than 2% (10). Another study found PPV for haemoptysis to be approx. 5% (men) (11).

In a survey of GPs’ assessment of lung cancer patients' symptoms at initial presentation, we have shown that only 33% had alarm symptoms (5). The remaining patients presented with what the GP characterised as serious general symptoms or vague/ill-defined symptoms. Futhermore, we have also shown (unpublished data) that GPs refer only 45% of the lung cancer patients with alarm symptoms to fast track. Coupled with the fact that GPs often consider the possibility of cancer in the consultation (approx. 7% of consultations) (12), this means that it can be assumed that the investigation of lung cancer in general practice will take longer because of an experience-based reluctance to refer to the fast track or to start the investigation of patients presenting with other symptoms. These strategies in general practice have only to a limited extent been explored in larger studies, and the exact use of imaging in the detection of lung cancer is unknown.

A chest x-ray is often the first choice in the investigation of lung cancer. It is easily accessible, inexpensive and shows tumours of 2-3 cm or larger if they are located in the lung parenchyma. Unfortunately, x-rays are less suitable for central and smaller tumours. It has been shown that between 15-23% of all lung cancer patients have a normal chest x-ray before the diagnosis is made (6;13). A false negative result has been shown to delay the diagnosis up to six times (14). On the other hand, we know that a chest CT scan is effective in the investigation of lung cancer with high sensitivity and reasonable specificity (15), which means that it can be used to detect patients with localised and potentially curable disease. However, a CT scan is more expensive, gives a significantly higher radiation dose and often results in finding nodules which then lead to several investigations that may ultimately prove unnecessary while having incurred costs for the patient and society.

Lyt

Læs fonetisk

Ordbog - [Vis detaljeret ordbog](http://www.google.com/dictionary?source=translation&hl=da&q=&langpair=da|en)

The idea of this project is to generate new knowledge of the way GPs interpret cancer patients' symptoms in a system with cancer fast tracks and how they use imaging. Furthermore, the idea is to investigate whether GPs would be more likely to refer for examination of lung cancer if they are offered the possibility of direct chest CT. Moreover, we want to evaluate the way lung cancer patients are examined through the fast track and the impact of chest CT before an evaluation by a chest physician.

**2. Hypothesis and purposes**

The project has three main hypotheses to be tested. Firstly, that the GPs’ use of the cancer fast track and examination for suspected lung cancer can be optimised in relation to the interpretation of symptoms and subsequent referral practices and radiological investigation. Secondly, that the GPs with special training in the diagnostic process can change the referral routines and that direct access to rapid CT scans leads to earlier diagnosis of lung cancer. Finally, that chest CT scans before referral to the Department of Pulmonary Medicine reduces delay and save the chest physician time.

**Purposes:**Lyt

Læs fonetisk

Ordbog - [Vis detaljeret ordbog](http://www.google.dk/dictionary?source=translation&hl=da&q=&langpair=)

**Section 1:**

1. To describe the way the GPs interpret the symptoms of their lung cancer patients and to describe the referral pathways for patients with alarm symptoms compared to patients with vague/ill-defined symptoms, with specific focus on the cancer fast track.
2. To describe which radiological examinations the patients are referred to through the diagnostic process.
3. To analyse the correlation between the GPs’ interpretations of symptoms, the way they refer the patients and the use of imaging in relation to delay.

**Section 2:**

1. In a two-arm, clinical, controlled, randomised trial with direct referral to CT scan compared to usual practice, to test:

A) The effect on time to diagnosis and TNM stage.

B) The effect on how CT is used in this group of patients and the overall change in the use of imaging and fast-track referrals.

1. As a further experiment to randomise all patients referred for the existing fast track to either direct CT scan of chest and upper abdomen or to evaluation by the chest physician, in order to test:
   A) Fast track performance measured by number of CT scans and chest physician specialist time per diagnosis, and whether there is a difference between the intervention and the control group.

1. Lyt
2. Læs fonetisk
4. Ordbog –
5. [**Vis detaljeret ordbog**](http://www.google.com/dictionary?source=translation&hl=da&q=&langpair=da|en)

**3. Methods**

### Data sources

***Section 1:*** The Research Unit for General Practice has established a research database with all newly diagnosed cancer patients from the Central Denmark Region and the Region of Southern Denmark during one year (2007-2008). It contains approx. 12,000 patients of whom 1,450 have primary lung cancer. Data is collected through questionnaires to GPs including the patients’ symptoms, time to diagnosis, referral patterns etc. Data will be linked with registry data on the patients, including:

The Civil Registration System: contains data on migration and death. The Danish Cancer Registry: contains information about all cancers, date of diagnosis, type of cancer and stage at diagnosis. The County Hospital Discharge Registry: includes data for each hospital admission and outpatient visit. All radiological procedures are listed here as well. The Danish Registry of Causes of Death: contains data on date, place and cause of death. Furthermore, we will use the Danish Lung Cancer Registry which contains information about examinations and delays.

***Section 2:*** We aim to evaluate the effect of a technological and organisational upgrade in the detection of lung cancer through carrying out a clinical, cluster-randomised, unblinded, two-arm (1:1) study where GPs get direct access to chest CT scans. Furthermore, we perform a 1:1 randomisation to either a primary CT scan or a chest physician for all patients referred through the usual fast track. All patients referred to the direct access CT scan group and patients from the lung cancer fast track are followed during the trial regarding diagnosis and further diagnostic investigations. The effect is measured on all lung cancer cases in the participating practices. Furthermore, we examine the overall use of x-rays and fast-track referrals. See Figure 1 for the study design diagram.

Data Collection: the patients diagnosed with ‘Cancer Pulmonis’ (International Classification of Diseases - ICD-10: C34.0-34.9) will be identified through the Patient Administration System (PAS). The GP with whom the patient is registered immediately receives a brief questionnaire developed at The Research Unit for General Practice. This questionnaire includes symptoms at presentation (alarm/vague symptoms), delays, co-morbidity, use of diagnostic tools and whether the patient was referred directly by fast track or not. The developed questionnaires will be piloted before use.

Cluster eligibility: all practices referring to the Department of Pulmonary Medicine, Aarhus University Hospital.

Exclusion of patients: former lung cancer patients.

Intervention: direct referral for CT scan. Control group continues with the usual referral pattern.

In the intervention group, GPs continue to refer to the lung cancer fast track when indicated. In addition, they are allowed to refer directly to a rapid chest CT scan in cases where they find reasons to examine the patient for lung diseases without having sufficient suspicion of lung cancer to refer the patient to the lung cancer fast track. The GPs will be offered special training related to the diagnosis of lung cancer in general practice before the new referral option is introduced. This will be done by offering training sessions and written material. The training will include risk groups, alarm symptoms, symptom complexes and interpretation of CT scan descriptions.

Materials: we co-operate with the Department of Radiology, Aarhus University Hospital who will carry out the CT scans in the same quality as for lung cancer patients already scanned in the fast track today. We plan to do a pilot test in the above-mentioned setting and, if necessary, involve an additional department to increase the strength.

Lyt

Læs fonetisk

Ordbog - [Vis detaljeret ordbog](http://www.google.com/dictionary?source=translation&hl=da&q=&langpair=da|en)

The project is designed and will be reported according to the CONSORT guidelines (16).

Lyt

Læs fonetisk

Ordbog - [Vis detaljeret ordbog](http://www.google.com/dictionary?source=translation&hl=da&q=&langpair=da|en)

**Analyses**

***Section 1:*** Questionnaire data will be combined with registry data for lung cancer patients and the data validated by the Danish Cancer Registry. We prepare descriptive statistics about symptom presentation, referral type and use of imaging in the investigation. When using multivariate analysis including survival analysis, it is possible to analyse the relationship between the GPs’ interpretation of symptoms, referral patterns and radiological investigation in relation to delay, cTNM stage and 1-year mortality.

***Section 2:*** The primary endpoint is delay which will be positively skewed. We will describe this delay by median and quartile values and apply waiting time analysis. Secondary endpoints are reference type (fast track), stage (TNM), primary use of CT and 1-year mortality. Mortality will be analysed with Cox's proportional hazard and spline models in crude and adjusted models. The data in the intervention and the control groups will mainly be analysed for differences between the two groups. We will be aware of clusters of patients in the same general practice if this occurs significantly. In addition, we will evaluate the use of imaging in the two groups of practices and for the two groups of cancer patients.

**Power, calculation and dimensioning**

The power calculation is based on data from our database on the pathway from first symptom to treatment and on data from the study of ‘Contact and Diagnosis Patterns in General Practice’. We assume that lung cancer is diagnosed in 20% of the patients referred to the fast track and in 5% of the patients referred through other pathways. The median time from first presentation to diagnosis of lung cancer is 33 days (2008).

We assume that the variation between and within practices in this study is roughly comparable because one GP will only diagnose one lung cancer patient a year on average. It is unlikely that lung cancer patients choose specific GPs. There could, however, be a higher incidence of cancer in some areas with many smokers and in practices with many elderly. To account for an unknown intra-cluster correlation coefficient (ICC), we count on a design effect of 1.25. The analysis will be adjusted for clusters.

Overall, PPV for haemoptysis (men) is approx. 5% and therefore presumably lower for less specific symptoms. Thus, in theory, GPs must refer at least 15 patients to find the one lung cancer patient and 5-8 of them should be referred through another pathway than the fast track. As the incidence of lung cancer is assumed to be 150/100,000 (40 + years old) (17), we can thus assume that GPs must refer approx. 2250/100,000 to some form of examination in order to diagnose these patients (see above). However, from current practice we know that approx. 685/100.000 are referred and of these, approx. 45% (n = 308/100,000) go through the fast track, while the rest (n = 377/100,000) go through other pathways. Since the study area includes a population of approx. 175.000 (40+ years old) (18), the average number of patients referred to the fast track will be approx. 539 and through other pathways 659. In the randomised trial we will thus have 230 referred for the intervention pathway in one year.

In a randomised study where we want to show a decrease in diagnostic delay from the current median of 33 days to a level where only 30% of the patients have to wait 33 days or more (one-sided alpha of 5% and power of 80%), we have to include 54 patients in each arm in a 1:1 randomisation. If we assume a design effect of 1.25, we have to include a total of 135 lung cancer patients. Approx. 250 lung cancer patients are diagnosed at the Department of Pulmonary Medicine, Aarhus University Hospital per year.

**4. Ethics and data management**

The National Board of Health will be applied for permission to use medical records from general practice and from hospital. We will apply to the Data Protection Agency for permission to establish a private research registry for CAPS, a comprehensive collection of registry data at The Research Unit for General Practice and Statistics Denmark. The Central Denmark Region Committees on Biomedical Research Ethics will be asked whether the project falls under the legislation of the committee, and we will secure necessary permits.
The Multipractice Study Committees of 1) the Organisation of General Practitioners in Denmark, DSAM and 2) the Danish College of General Practitioners in Denmark, PLO are asked to recommend practitioners to participate in the randomised trial. At the same time, we apply for salary for data collection in general practice. The project will be conducted according to good clinical practice for randomised controlled trials and will be mandatory indexed at www.clinicaltrials.gov.

**5. Supervision, agreements and feasibility**

A workstation will be available at The Research Unit for General Practice which ensures a close connection with an active research group, access to relevant statistical programs and participation in relevant courses and study groups. Supervision will run regularly under the PhD programme.
Contracts: we have an agreement with the Department of Radiology, Aarhus University Hospital about CT scans and descriptions. In addition, agreements are made with the Department of Pulmonary Medicine, Aarhus University Hospital for help with the randomisation of patients referred for fast track and assessment of patients. CaP has earlier made an agreement with the Central Denmark Region for support in clinical trials.

**6. Time schedule**

The study is planned as a three year PhD programme:
Year 1: Planning and start of the RCT project, training of doctors. Participation in mandatory and relevant PhD courses.
Year 2: Completion of Section 1. Completion of data collection, registration.
Year 3: Data processing, completion of Section 2 and publication. Preparation of PhD dissertation.

**7. Publication of results**

The results will be published as four articles in international, peer-reviewed journals with Louise Mahncke as first author and the supervisors as co-authors meeting the criteria for co-authorship. The study will be summarised in a PhD thesis. Furthermore, the results will be disseminated through oral presentations and posters at scientific symposia and conferences.

**8. Experience of applicant**

The applicant is an MD, graduated from Aarhus University (January 2006). The last 2.5 years she has been working at the Department of Oncology, Aarhus University Hospital. She has attended courses relevant to the project, including GCP and "Clinical trials in Oncology”. Prior to the project she participated in the idea and design phase, protocol draft and permissions.

**9. Budget**

The full amount for the project is 2.605.727 DKK, including 1.649.796 DKK which is salary for PhD student Louise Mahncke, 515.282 DKK for technical administrative staff, and 362.300 DKK for other expenses.

**10. Perspectives**

The study will provide new knowledge of the way GPs interpret symptoms, the way they refer patients with suspected cancer or other lung diseases, and their use of diagnostic imaging and cancer fast track pathways. Furthermore, it will clarify the effects of referral patterns on system delay and ultimately on the survival from cancer. It will also provide a unique opportunity to gather the necessary knowledge about the effects of direct referral for fast CT scan, and it might be a decision aid as to whether to introduce direct referral for CT scans in general practice for a group of patients. The study will provide new knowledge about which diagnostic examination results in the shortest delay and, thus, in the best result for the treatment of lung cancer.

**References**

(1) Dansk Lunge Cancer Gruppes Årsrapport 2009. DLCG 2011 Available from: URL: [www.lungecancer.dk](http://www.lungecancer.dk)

(2) Coleman MP, Forman D, Bryant H, Butler J, Rachet B, Maringe C, et al. Cancer survival in Australia, Canada, Denmark, Norway, Sweden, and the UK, 1995-2007 (the International Cancer Benchmarking Partnership): an analysis of population-based cancer registry data. Lancet 2011 Jan 8;377(9760):127-38.

(3) Storm HH, Engholm G, Hakulinen T, Tryggvadottir L, Klint A, Gislum M, et al. Survival of patients diagnosed with cancer in the Nordic countries up to 1999-2003 followed to the end of 2006. A critical overview of the results. Acta Oncol 2010 Jun;49(5):532-44.

(4) Rogers TK. Primary care radiography in the early diagnosis of lung cancer. Cancer Imaging 2010 Mar 16;10:73-6.:73-6.

(5) Nielsen TN, Hansen RP, Vedsted P. Præsentation af symptomer i almen praksis hos patienter med cancer [Symptom presentation in cancer patients in general practice]. Ugeskr Laeger 2010;172(41):2827-31.

(6) Bjerager M, Palshof T, Dahl R, Vedsted P, Olesen F. Delay in diagnosis of lung cancer in general practice. Br J Gen Pract 2006 Nov;56:863-8.

(7) Olesen F, Hansen RP, Vedsted P. Delay in diagnosis: the experience in Denmark. British Journal of Cancer 2009;101(Suppl 2):S5-S8.

(8) Pakkeforløb lungekræft. DSAM 2011 Available from: URL: [www.dsam.dk](http://www.dsam.dk)

(9) Svendsen RP, Stovring H, Hansen BL, Kragstrup J, Sondergaard J, Jarbol DE. Prevalence of cancer alarm symptoms: A population-based cross-sectional study. Scand J Prim Health Care 2010;28(3):132-7.

(10) Hamilton W, Peters TJ, Round A, Sharp D. What are the clinical features of lung cancer before the diagnosis is made? A population based case-control study. Thorax 2005 Dec;60(12):1059-65.

(11) Jones R, Charlton J, Latinovic R, Gulliford M. Alarm symptoms and identification of non-cancer diagnoses in primary care: cohort study. BMJ 2009;339:1-9.

(12) Moth G, Vedsted P, Olesen F, Christensen B. Hvad går folk egentlig til læge med? Danmarks Læger 2009;1(3):9.

(13) Quekel LG, Kessels AG, Goei R, van Engelshoven JM. Miss rate of lung cancer on the chest radiograph in clinical practice. Chest 1999 Mar;115(3):720-4.

(14) Bjerager M. Delay in diagnosis and treatment of lung cancer [thesis]. 1 ed. Aarhus: Research Unit and Department of General Practice, Faculty of Health Sciences, University of Aarhus; 2006.

(15) Schneider J. Early detection of lung cancers - Comparison of computed tomography, cytology and fuzzy-based tumor markers panels. Cancer Biomark 2010;6(3-4):149-62.

(16) CONSORT Statement Website. <http://www.consort-statement.org/> . 22-8-2007.
Ref Type: Internet Communication

(17) Det Nationale Indikatorprojekt. NIP - Det Nationale Indikatorprojekt. <http://www.nip.dk/> . 15-8-2006.
Ref Type: Internet Communication

(18) Danmarks statistik. Statistikbanken. [www.statistikbanken.dk](http://www.statistikbanken.dk) . 1-1-2005.
Ref Type: Internet Communication

Figure 1. The study outline and flow for entire randomised, controlled trial on the effect of direct, fast access to CT scan for patients with suspected lung cancer.
